# HDAC6 inactivates Runx2 promoter to block osteogenesis of bone marrow stromal cells in age-related bone loss of mice

**DOI:** 10.1186/s13287-021-02545-w

**Published:** 2021-08-28

**Authors:** Chao Ma, Juan Gao, Jun Liang, Weixiang Dai, Zhenfei Wang, Mengjiao Xia, Tao Chen, Sen Huang, Jian Na, Long Xu, Shiming Feng, Kerong Dai, Guangwang Liu

**Affiliations:** 1 Department of Orthopedic Surgery, Xuzhou Central Hospital, Xuzhou Clinical School of Xuzhou Medical University, The Affiliated XuZhou Hospital of Medical College of Southeast University, Xuzhou Clinical Medical College of Nanjing University of Chinese Medicine, Xuzhou, 221009 Jiangsu China; 2 Department of Gynaecology and Obstetrics, Xuzhou Central Hospital, Xuzhou Clinical School of Xuzhou Medical University, The Affiliated XuZhou Hospital of Medical College of Southeast University, Xuzhou Clinical Medical College of Nanjing University of Chinese Medicine, Xuzhou, 221009 Jiangsu China; 3 Department of Endocrinology, Xuzhou Central Hospital, Xuzhou Clinical School of Xuzhou Medical University, The Affiliated XuZhou Hospital of Medical College of Southeast University, Xuzhou Clinical Medical College of Nanjing University of Chinese Medicine, Xuzhou, 221009 Jiangsu China; 4 Shanghai Key Laboratory of Orthopaedic Implants, Department of Orthopaedic Surgery, Shanghai Ninth People’s Hospital, Shanghai Jiaotong University School of Medicine, Shanghai, 200011 China

**Keywords:** Aging, Osteoporosis, Bone marrow stromal cells, Runx2, Histone deacetylases 6, Androgen receptor

## Abstract

**Background:**

Senile osteoporosis can cause bone fragility and increased risk for fractures and has been one of the most prevalent and severe diseases affecting the elderly population worldwidely. The underlying mechanisms are currently intensive areas of investigation. In age-related bone loss, decreased bone formation overweighs increased bone resorption. The molecular mechanisms underlying defective bone formation in age-related bone loss are not completely understood. In particular, the specific role of histone acetylation in age-related bone loss has not been examined thoroughly.

**Methods:**

We employed 6- and 18-month-old mice to investigate the mechanisms of defective bone formation in age-related bone loss. Bone marrow stromal cells (BMSCs) were induced to undergo in vitro osteogenic differentiation. Chromatin immunoprecipitation (ChIP) was used to investigate the binding of histone deacetylases (HDACs) on Runx2 promoter in BMSCs. Luciferase reporter and transient transfection assay were employed to study Runx2 gene expression modulation by HDAC and androgen receptor (AR). siRNA and HDAC6 inhibitor, Tubastatin A, were used to inhibit HDAC6 in vitro. And systemic administration of Tubastatin A was used to block HDAC6 in vivo.

**Results:**

Age-related trabecular bone loss was observed in 18-month-old mice compared with 6-month-old mice. In vitro osteogenic differentiation potential of BMSCs from 18-month-old mice was weaker than 6-month-old mice, in which there was Runx2 expression inactivation in BMSCs of 18-month-old mice compared with 6-month-old mice, which was attributable to HDAC6-mediated histone hypoacetylation in Runx2 promoter. There was competitive binding of HDAC6 and AR on Runx2 promoter to modulate Runx2 expression in BMSCs. More importantly, through siRNA- or specific inhibitor-mediated HDAC6 inhibition, we could activate Runx2 expression, rescue in vitro osteogenesis potential of BMSCs, and alleviate in vivo age-related bone loss of mice.

**Conclusion:**

HDAC6 accumulation and histone hypoacetylation on Runx2 promoter contributed to the attenuation of in vitro osteogenic differentiation potential of BMSCs from aged mice. Through HDAC6 inhibition, we could activate Runx2 expression and osteogenic differentiation potential of BMSCs from aged mice and alleviate the age-related bone loss of aged mice. Our study will benefit not only for understanding the age-related bone loss, but also for finding new therapies to treat senile osteoporosis.

**Supplementary Information:**

The online version contains supplementary material available at 10.1186/s13287-021-02545-w.

## Introduction

Age-related bone loss in elderly people, a disease known as senile osteoporosis, is associated with human aging. It can cause bone fragility and increased risk for fractures. It has been one of the most prevalent and severe diseases affecting the elderly population worldwidely and a public health problem [1]. The cellular and molecular mechanisms underlying age-related bone loss are currently intensive areas of investigation with the aim of developing new approaches to prevent and treat it in elderly people [1].

As a hard tissue, bone comprises bone matrix and bone cells. Among bone cells, the most important two types of bone cells are osteoclasts and osteoblasts [2]. Osteoclasts, derived from the monocytic hematopoietic lineage, are responsible for bone resorption and initiate bone remodeling. In contrast, osteoblasts, originated from bone marrow stromal cells (BMSC), are able to synthesize new osteoid and to complete its mineralization. Under physiological conditions, the amount of bone removed by the osteoclasts remains relatively equal to the amount of bone formed by the osteoblasts. In constrast, this is not the case during aging. Increased bone resorption and/or decreased bone formation can result in bone loss. More importantly, the previous study demonstrated that bone formation decrease but not bone resorption increase is likely to be the principal pathophysiological mechanism underlying age-related bone loss [3].

Bone formation depends on the amount and activity of osteoblasts during bone remodeling. Osteoblasts are differentiated from osteoprogenitor and stem cells present in the non-hematopoietic compartment of bone marrow (known as bone marrow stroma cells, BMSCs) [4]. BMSCs osteogenic differentiation involves preosteoblasts, osteoblasts, mature osteoblasts, and ultimately the deposition and mineralization of the extracellular matrix. Several transcription factors have been identified to control and modulate BMSCs osteogenic differentiation. Of these, runt-related transcription factor 2 (Runx2) plays an essential role. The onset of osteogenic differentiation is marked by Runx2 expression upregulation [5]. Alkaline phosphatase (ALP) is expressed during the early stages of osteogenesis, and osteopontin (Opn) and osteocalcin (Ocn) are expressed in more mature osteoblasts during matrix maturation and mineralization [6]. Investigating the Runx2 expression regulation mechanisms is of importance to our insights into BMSCs osteogenesis and the application of BMSCs to treat human diseases.

Chromatin is composed of double helix DNA with core histones, and the latter are potential targets of post-translational modifications [7]. These histone modifications could modulate gene transcription by changing chromatin structure [8, 9] or by providing binding platforms for other transcriptional regulators [10, 11]. Of these histone modifications, acetylation is generally associated with loose chromatin and active gene transcription [9]. Acetyl groups are added by histone acetyltransferases (HATs) and removed by histone deacetylases (HDACs).

The molecular mechanisms underlying defective bone formation in age-related bone loss are not completely understood. The specific role of histone acetylation in age-related bone loss has not been examined thoroughly, although over the last decade, it has been studied in diverse cellular processes including mitosis, programmed cell death and oncogenesis. We even have no idea how to prevent or treat bone loss in senile osteoporosis through modulating BMSCs. In the current study, we employed 6- and 18-month-old mice to investigate the mechanisms of defective bone formation in age-related bone loss. The results revealed Runx2 expression inactivation in BMSCs of 18-month-old mice compared with 6-month-old mice, which was attributable to HDAC6-mediated histone hypoacetylation in Runx2 promoter. We further observed that there was competitive binding of HDAC6 and androgen receptor (AR) on Runx2 promoter to modulate Runx2 expression in BMSCs. More importantly, through siRNA- or specific inhibitor-mediated HDAC6 inhibition, we could activate Runx2 expression, rescue in vitro osteogenesis potential of BMSCs, and alleviate age-related bone loss of mice.

## Materials and methods

### Animals

Male C57BL/6 mice (SIPPR-BK Laboratory Animal Co. Ltd, Shanghai, China) were housed under SPF conditions. All animal operations were approved by the Animal Ethics Committee of Xuzhou Central Hospital (Animal Protocol No. XZZX-LJ-20190201-18)

12 animals were included per group. Animals were euthanized using isoflurane inhalation anesthesia followed by cervical dislocation. The right femur, tibia and lumbar vertebrae (L5) were prepared for micro computed tomography (micro-CT) scan; the left femur and tibia were used for BMSCs isolation. Peripheral serum was taken for the measurement of osteocalcin and *N*-telopeptide (Biomedical Technologies, Stoughton, MA).

Animals were administrated intraperitoneally with tubastatin A (Sigma Chemical Co., St. Louis, MO) in 0.9% saline with 4% DMSO and 30% PEG300 at dose of 8 mg/kg once every two days for 30 days. After 30 days, animals were euthanatized for micro-CT and serum biomarkers analysis.

### Micro-CT

Femurs, tibias, and lumbar vertebrae were harvested and preserved in 70% ethanol. Bones were scanned using a micro-CT instrument (μCT-80, Scanco Medical AG, Bassersdorf, Switzerland). Standard nomenclature and guidelines for assessment of bone microstructure were followed, as recommended by the American Society for Bone and Mineral Research [12]. The bones were scanned at a low resolution, an energy level of 55 kVp, intensity of 145 μA, and a fixed threshold of 220. Trabecular bone volume fraction and microarchitecture of the distal femur, proximal tibia and L5 vertebrae were evaluated in the secondary spongiosa, starting proximately at 0.6 mm distal to the growth plate, and extending distally 1.5 mm. Approximately 230 consecutive slices were made at 10.5 μm interval at the distal end of the growth plate and extending in a proximal direction, and 100 contiguous slices were selected for analysis. The main bone parameters are BV/TV (the relative volume of calcified tissue in the selected volume of interest (VOI), Tb.N (the number of trabeculae), and Tb.Sp (trabecular separation; a measurement of the thickness of the spaces between the trabeculae, inversely proportional to the trabecular thickness).

### BMSCs isolation, in vitro culture and osteogenesis

The bone marrow from femur and tibia were suspended in cold PBS with 2% FBS and passed through a 70 μm filter. Filtered bone marrow cells were suspended in PBS with 2% FBS and 0.1 g/L phenol red and then enriched for lineage negative (Lin−) cells using the SpinSep system (Stem Cell Technologies, Vancouver, BC, Canada). The cells were incubated with a murine progenitor enrichment cocktail (Stem Cell Technologies) on ice for 30 min, washed, and then incubated with dense particles on ice for 20 min. The cells were then centrifuged at 1200*g* for 10 min, and the cells at the density medium/PBS interface were collected.

Enriched MSCs were seeded onto culture plates at a density of 0.1 × 10^6^ cells/cm^2^ in α-MEM containing 100 units/ml penicillin (Gibco) and 100 μg/ml streptomycin (Gibco). The media were changed after 72 h and adherent cells were maintained in culture with twice weekly media changes.

To induce osteogenic differentiation, BMSCs were treated with 100 nM dexamethasone, 10 mM β-glycerophosphate disodium and 50 μg/ml ascorbic acid.

BMSCs were treated in vitro with tubastatin A (Sigma) in DMSO at 8 μM for 21 days. Vehicle controls were treated with culture medium with DMSO only.

### Quantification of CFU-osteoblasts (CFU-OB)

BMSCs were seeded at a density of 3 × 10^6^ cells/10 cm^2^ well and maintained for 28 days in α-MEM containing 15% FBS, 50 mM ascorbic acid, and 10 mM β-glycerophosphate (Sigma) with one-half of the medium replaced every 3d. After fixation in 50% ethanol and 18% formaldehyde, the cultures were stained using Von Kossa’s method to visualize and quantify the number of colonies containing mineralized bone matrix.

### ALP staining and quantification

BMSCs were washed with PBS and fixed with 4% paraformaldehyde for 10 min at 4 °C. Then the cells were incubated in 0.1% naphthol AS-MX phosphate (Sigma) and 0.1% fast red violet LB salt (Sigma) in 2-amino-2-methyl-1,3-propanediol (Sigma) for 10 min at room temperature, washed with PBS, and then observed under a digital camera.

For ALP quantification, BMSCs were washed with PBS and then scraped into ddH2O. Three cycles of freezing and thawing were performed. ALP activity was determined at 405 nm using p-nitrophenyl phosphate (pNPP) (Sigma) as the substrate. Total protein content was determined with the BCA method, read at 562 nm and calculated according to a series of albumin (BSA) standards. ALP levels were normalized to the total protein content at the end of the experiment.

### Alizarin red staining and quantification

BMSCs were washed with PBS and fixed with 4% paraformaldehyde for 10 min at 4 °C. After fixation, the cells were washed with PBS and incubated in 40 mM Alizarin red (pH 4.2) for 30 min at 37 °C, then washed with PBS and imaged.

Decalcification was performed using 0.1 M HCl overnight at 4 °C. Then, 20 μL of samples were transferred to the test tubes containing 1 mL of methyl thymol blue solution and 1 mL of alkaline solution. Absorbance was determined at 610 nm.

### RT-PCR

Total RNA was isolated from cultured cells using RNeasy Mini Kit (Qiagen, Valencia, CA, USA) in accordance with the manufacturer’s protocol. For RT-PCR, single-stranded cDNA was reverse transcribed from 1 μg total RNA using reverse transcriptase with oligo-dT primer. Quantitative PCR analysis was performed on a 96-well plate ABI Prism 7500 Sequence Detection system (Applied BioSystems, Foster City, CA, USA) using SYBR Green PCR Master Mix (Takara Bio Inc., Otsu, Japan). Cycling conditions was as follows 94 °C, 5secs; 60 °C, 34secs; and 72 °C for 40 cycles. β-actin was used as internal control. Primer sequences were shown in Additional file 1: Table S1.

### Western blot

Histone preparations were made from young and aged mice BMSCs. Equal amounts of core histones were resolved by 15% SDS-PAGE, transferred onto PVDF membranes and probed with the following antibodies: acetylated H3K9/K14 (#9677, Cell Signaling Technology, Danvers, MA, USA), acetylated H4K12 (#13944, Cell Signaling Technology, Danvers, MA, USA), HDAC1 (#34589, Cell Signaling Technology, Danvers, MA, USA), HDAC3 (#85057, Cell Signaling Technology, Danvers, MA, USA), HDAC4 (MA5-15580, ThermoFisher scientific), HDAC5 (#98329, Cell Signaling Technology, Danvers, MA, USA), HDAC6 (PA1-41056, ThermoFisher scientific), total H3 (#4499, Cell Signaling Technology, Danvers, MA, USA), total H4 (#13919, Cell Signaling Technology, Danvers, MA, USA) and β-actin (#4970, Cell Signaling Technology, Danvers, MA, USA).

### Chromatin immunoprecipitation (ChIP)

Cells were cross-linked with 1% formaldehyde for 10 min at 37 °C and crude nuclei were purified. The crude nuclei were sonicated to produce chromatin fragments of approximately 500 bp. The antibodies used in the ChIP assay were described in Western blot. Rabbit IgG (Sigma) was included as a negative control. For each ChIP assay, 2–5 μg of antibodies were added and the samples were incubated overnight at 4 °C. Primers targeting the regions 1 kb (− 1 kb) and 0.2 kb (− 0.2 kb) upstream of the transcription start site (TSS) of Runx2 gene were used. Primer sequences used in ChIP-qPCR were listed in Additional file 1: Table S2. ChIP regions were displayed in Additional file 1: Figure S1.

### Amplification of Runx2 promoter region and construction of 5’ deletion mutants

A 1550 bp (− 1280 bp/ + 270 bp) fragment of Runx2 promoter was amplified from C3H10T1/2 cells with pfu DNA polymerase (Takara) with addition of two restriction sites: XhoI (5’) and Hind III (3’). This XhoI/Hind III fragment was cloned into the plasmid pGL3-Basic (Promega, CharbonniËres, France), named − 1280 bp/ + 270 bp. The 5’ deletion constructs − 990 bp/ + 270 bp, − 694 bp/ + 270 bp, − 347 bp/ + 270 bp, − 7 bp/ + 270 bp, − 347 bp/− 1 bp, − 327 bp/− 1 bp, − 62 bp/− 1 bp, and − 9 bp/− 1 bp were created by PCR using the − 1280 bp/ + 270 bp as template.

### Luciferase reporter assay

Cells were seeded into 24-well plates. All plasmids for transfection were isolated by QIAGEN plasmid purification kit (QIAGEN). Transient transfection by lipofectamine2000 (Invitrogen Carlsbad, CA, USA) were performed according to the manufacturer’s instruction and phRL-SV40 vector (Promega, Madison, WI, USA) was used as transfection efficiency control. Forty-eight hours after transfection, both firefly and renilla luciferase activities were measured using Dual-luciferase reporter assay system (Promega) with Luminoskan TL plus Luminometer (MTX, Labsystem, Vienna, VA, USA). The ratio of firefly’s activity to renilla was then obtained.

### RNAi

Plasmid vector loading shRNAs against HDAC6 for lentivirus packaging were purchased from Genecopoeia (Rockville, MD, USA). 293T cells were plated and the transfection complex was added to the culture medium at 293T 70–80% confluence. Then the cells were incubated in a CO_2_ incubator at 37 °C for 48 h and the medium was then collected. Lentivirus titer was evaluated by Lenti-X p24 Rapid titer kit (Clontech, Mountain View, CA, USA). After infection of BMSCs by lentivirus loading shRNAs against HDAC6, RT-PCR was perormed to analyze HDAC6 expression.

### Statistics analysis

Statistical significance was calculated by Student’s t*-*test for two-sample comparisons and one- and two-way ANOVA was used for multiple comparisons in software SPSS 16.0. Holm-Sidak method was used to find significant differences in ANOVA. *p* < 0.05 were defined as significant. All data are presented as mean ± SD unless otherwise specified. All data were obtained from at least three independent experiments.

## Results

### Age-related bone loss in mice

The peak bone mass of mice is reached between 5 and 6 month of age [13]. Therefore, 6-month-old mice were defined as “young” mice, and 18-month-old mice were defined as “aged” mice in the current study. Femurs, tibias, and L5 vertebrae were collected from young and aged mice respectively. Micro-CT was employed to assess the trabecular bone microstructure.

**Fig. 1:**
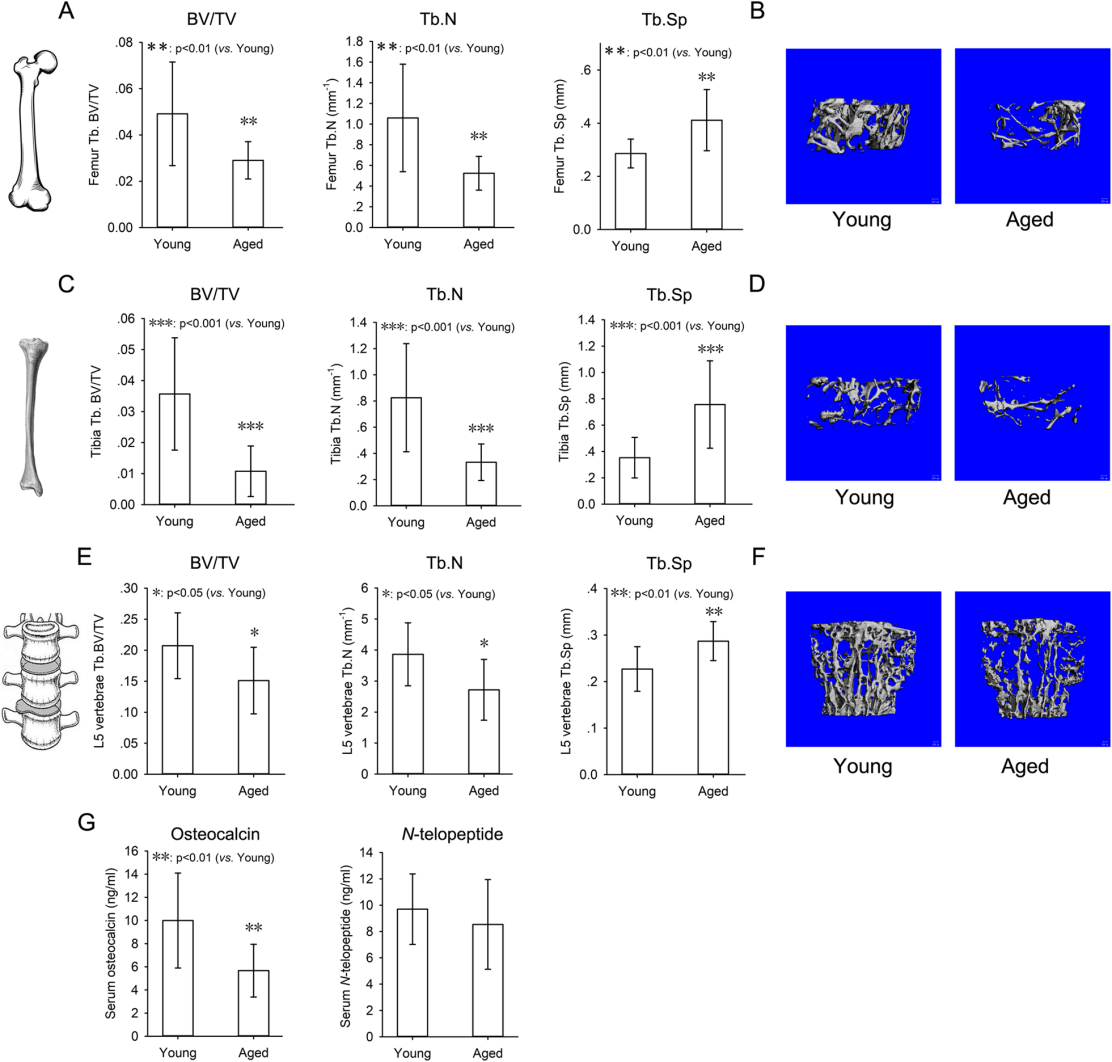
Age-related bone loss in mice. Femur (**A**), tibia (**C**) and L5 vertebrae (**E**) were harvested from 6- (Young) and 18-month-old (Aged) mice. Micro-CT was employed to evaluate the trabecular bone microstructure. Trabecular bone parameters including BV/TV, Tb.N, and Tb.Sp were quantified according to Micro-CT scans. Representative 3D reconstruction images of trabecular bone in the distal femur (**B**), proximal tibia (**D**), and L5 vertebrae (**F**) were displayed. Serum levels of bone turnover markers, osteocalcin and *N*-telopeptide, in young and aged mice were determined by ELISA (**G**). Data were shown as the means ± SD. *p < 0.05, **p < 0.01, ***p < 0.001, aged versus young

There was a 33% decrease in BV/TV, a 22% decrease in Tb.N and a 20% increase in Tb.Sp in the distal femur of aged mice compared with young mice (Fig. 1A). There was a 42% decrease in BV/TV, a 33% decrease in Tb.N and a 48% increase in Tb.Sp in the proximal tibia of aged mice compared with young mice (Fig. 1C). There was a 64% decrease in BV/TV, a 25% decrease in Tb.N and a 22% increase in Tb.Sp in the L5 vertebrae of aged mice compared with young mice (Fig. 1E). Micro-CT 3-D reconstruction images of trabecular bone also showed the same pattern (Fig. 1B, D, F).

Besides micro-CT scan, bone formation and resorption markers in serum were analyzed by ELISA to determine the bone turnover status of young and aged mice. There was no significant change in *N*-telopeptide, a marker of bone resorption, between young and aged mice (Fig. 1G). However, a significant decrease in osteocalcin, a marker of bone formation, was observed in serum from aged mice compared with young mice (Fig. 1G), suggesting that bone formation attenuated significantly in aged mice.

### Attenuation of in vitro osteogenesis potential of BMSCs from aged mice

The results described above revealed the age-related bone loss of aged mice. More importantly, the serum bone turnover markers analysis suggested that there was not bone resorption increase, but bone formation decrease in aged mice. So BMSCs were harvested from young and aged mice and cultured in vitro in the current study.

**Fig. 2:**
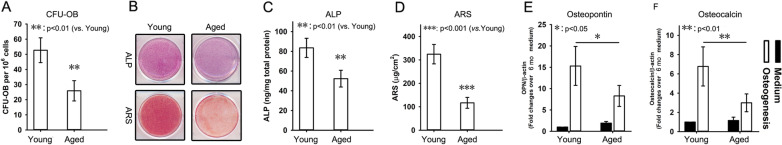
Attenuation of in vitro osteogenesis potential of BMSCs from aged mice. BMSCs were isolated from 6- (Young) and 18-month-old (Aged) mice respectively and cultured in vitro. The number of CFU-OB was quantified (**A**). The results are expressed as the number of CFU-OB per 10^6^ cells. BMSCs were then induced to undergo osteogenesis differentiation. Alkaline phosphatase (ALP) and alizarin red staining (ARS) were performed at weeks 1 and 3, respectively (**B**). The quantification of ALP (**C**) and alizarin red (**D**) were also performed. At week 2, total RNA was extracted and subjected to quantitative RT-PCR analysis using primers for osteopontin (**E**) and osteocalcin (**F**). β-actin was used as an internal control. Data were shown as the mean ± SD. *p < 0.05, **p < 0.01, ***p < 0.001, aged versus young

In BMSCs cultures from aged mice, there were 51% fewer CFU-OBs than from young mice (Fig. 2A). The BMSCs were induced to undergo osteogenic differentiation in vitro. At week 1, ALP staining revealed that ALP activation was weaker in the BMSCs from aged mice compared with young mice (Fig. 2B). At week 3, Alizarin Red staining in BMSCs from aged mice was much weaker than young mice (Fig. 2B). ALP (Fig. 2C) and ARS (Fig. 2D) quantification revealed the same pattern as staining. Total RNA was extracted at week 2 and subjected to quantitative RT-PCR to determine the expression of osteogenic markers. BMSCs from aged mice showed lower levels of osteopontin (Fig. 2E) and osteocalcin (Fig. 2F) mRNA than young mice in response to osteogenesis induction.

### HDAC6 accumulation and histone hypoacetylation on Runx2 promoter in BMSCs from aged mice

**Fig. 3:**
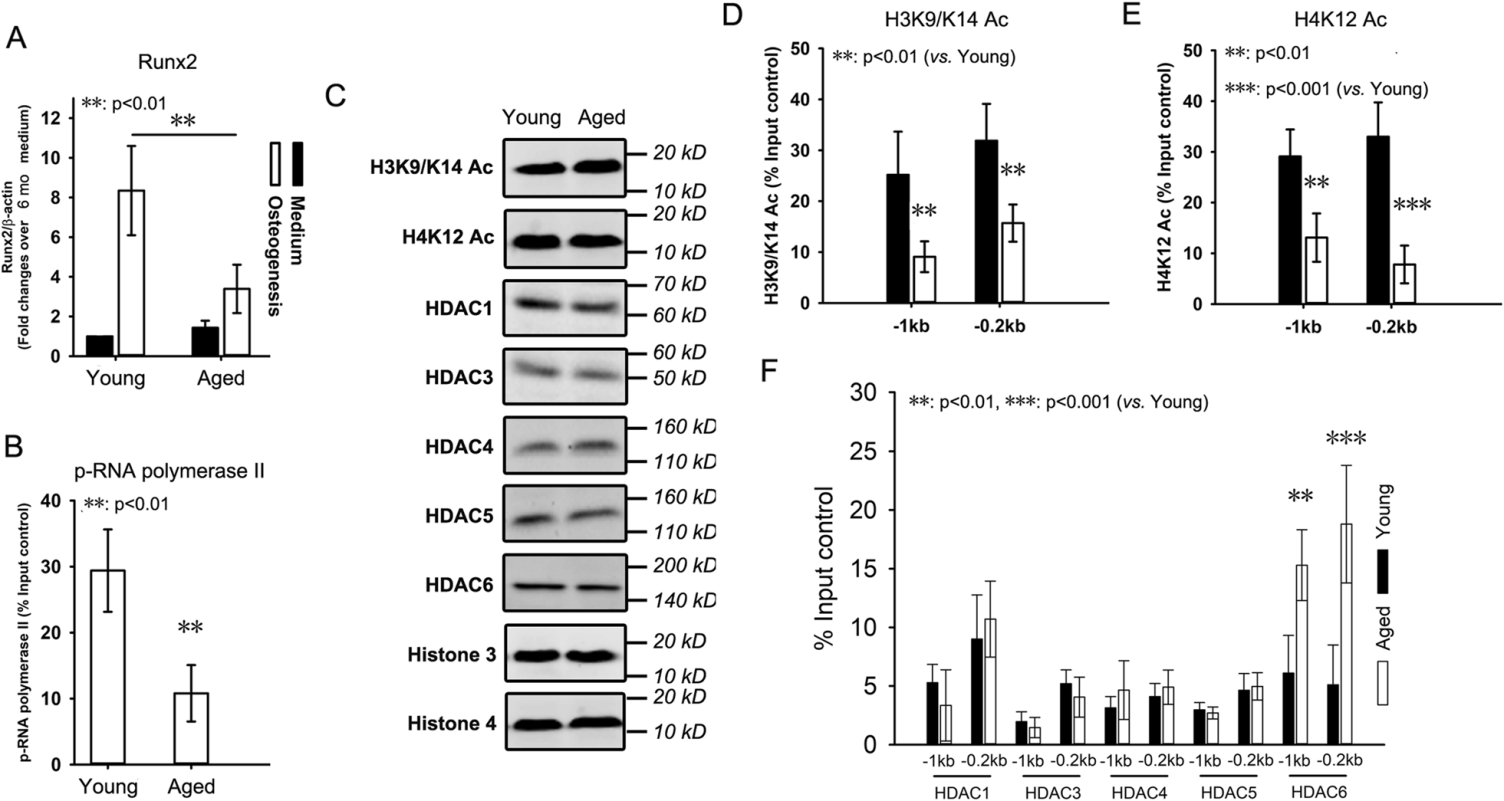
HDAC6 accumulation and histone hypoacetylation on Runx2 promoter in BMSCs from aged mice. BMSCs were isolated from 6- (Young) and 18-month-old (Aged) mice respectively and induced to undergo osteogenesis differentiation. At week 1, total RNA was extracted and subjected to quantitative RT-PCR analysis using primers for Runx2 (**A**). β-actin was used as an internal control. ChIP was employed to evaluate phosphorylated RNA polymerase II occupancy of the Runx2 promoter in young and aged BMSCs with osteogenic induction (**B**). Histone samples were collected. Global histone modification levels including acetylated H3K9/K14 and H4K12, and HDAC family members were determined by western blot (**C**). Histone 3 and 4 were used as loading controls. ChIP assays were used to examine acetylated H3K9/K14 (**D**)**,** acetylated H4K12 (**E**), and HDACs (**F**) occupancy in the Runx2 − 1 kb and − 0.2 kb promoter regions in young and aged BMSCs. The results were normalized to the percentage of various histone modifications in the input control. Data are shown as the mean ± SD. **p < 0.01, ***p < 0.001, aged versus young

Having observed the attenuation of osteogenic differentiation potential in BMSCs from aged mice, we aimed to elucidate the underlying mechanisms. BMSCs were induced to undergo osteogenic differentiation in vitro. At week 1, total RNA was extracted and subjected to quantitative RT-PCR to determine the expression of Runx2. The results revealed much lower level of Runx2 mRNA in response to osteogenesis induction in BMSCs from aged mice than young mice (Fig. 3A), suggesting that Runx2 in the BMSCs from aged mice was in an inactivation state in response to osteogenesis induction. Also, the results from ChIP revealed weaker binding of p-RNA polymerase II, a key component of transcription machinery on Runx2 promoter in BMSCs from aged mice compared with young mice (Fig. 3B).

Histone acetylation in Runx2 promoter was investigated in BMSCs from young and aged mice. We first examined the global expression levels of acetylated H3K9/K14 and H4K12, HDAC1, HDAC3, HDAC4, HDAC5, and HDAC6. Total H3 and H4 were used as loading controls. As shown in Fig. 3C, the global levels of all of the histone modifications examined remained stable in BMSCs from young and aged mice, suggesting that these histone modifications are not globally affected in the cellular level by mice age.

Then histone acetylation status on Runx2 promoter was investigated. We selected H3K9/K14 and H4K12 acetylation to represent the acetylation of H3 and H4, respectively. Two regions were examined: 1 kb and 0.2 kb upstream of the TSS of Runx2 (Additional file 1: Fig. S1). The ChIP results revealed significant decreases in H3K9/K14 (Fig. 3D) and H4K12 (Fig. 3E) acetylation within these two regions of Runx2 promoter in BMSCs from aged mice compared with young mice.

We then analyzed the binding of HDAC family members on Runx2 promoter. There were no differences in HDAC1, 3, 4, and 5 binding on Runx2 promoter in BMSCs from young and aged mice (Fig. 3F). In contrast, remarkable HDAC6 accumulation on Runx2 promoter was observed in BMSCs from aged mice compared with young mice (Fig. 3F).

### Competitive binding of HDAC6 and AR on Runx2 promoter to modulate Runx2 expression in BMSCs

**Fig. 4:**
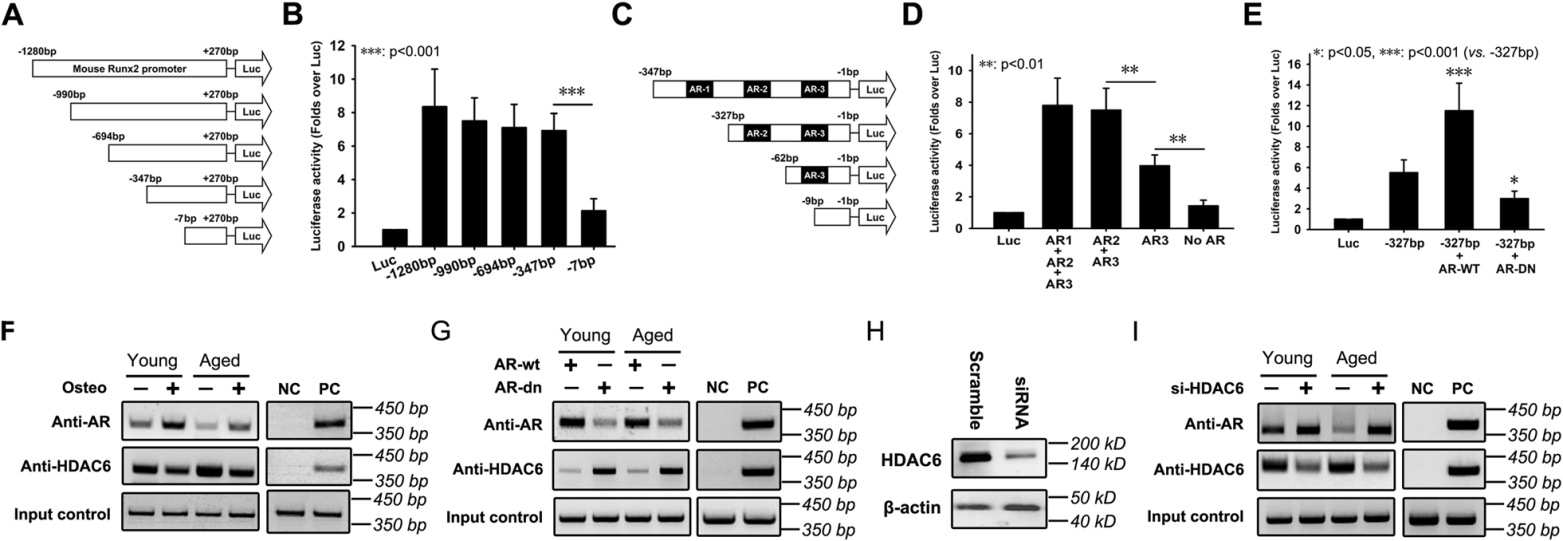
Competitive binding of HDAC6 and AR on Runx2 promoter to modulate Runx2 expression in BMSCs. Runx2 promoter (− 1280 bp ~  + 270 bp) deletion mutant-driven luciferase vectors were established (**A**). Luciferase activity of every vector was measured by transient reporter assay in BMSCs from young mice (**B**). Runx2 promoter (− 347 bp ~ − 1 bp) deletion mutant-driven luciferase vectors were established (**C**). Luciferase activity of every vector was measured in BMSCs young mice (**D**). Luciferase activity of Runx2 promoter (− 327 bp ~ − 1 bp)-driven reporter vector was measured in response to AR-WT or AR-DN co-transfection (**E**). ChIP was employed to evaluate AR and HDAC6 occupancy of the Runx2 proximal promoter in BMSCs from young and aged mice with and without osteogenic induction (**F**). ChIP was employed to evaluate AR and HDAC6 occupancy of the Runx2 proximal promoter in response to AR-WT or AR-DN overexpression (**G**). Lentivirus vector loading siRNA against HDAC6 was employed to knockdown HDAC6 expression (**H**). ChIP was employed to evaluate AR and HDAC6 occupancy of the Runx2 proximal promoter in response to HDAC6 knockdown (**I**). Luc: luciferase, AR: androgen receptor, AR-WT: wild type AR, AR-DN: dominant negative AR, PC: positive control, NC: negative control. In transient reporter assay, the results were expressed as fold changes in relative luciferase unit (RLU) of every vector relative to empty luciferase vector. Data are shown as the mean ± SD. *p < 0.05, **p < 0.01, ***p < 0.001

We amplified the mouse Runx2 promoter − 1280 bp/ + 270 bp by PCR and generated four deletion constructs, − 990 bp/ + 270 bp, − 694 bp/ + 270 bp, − 347 bp/ + 270 bp, and − 7 bp/ + 270 bp. The various Runx2 promoter fragments were cloned into pGL3-basic vector (Fig. 4A). Then transient reporter assay was performed in BMSCs from young mice in response to 48-h osteogenesis induction. The activity of four constructs exhibited significant increases compared with empty luciferase vector (Fig. 4B). However, there was a remarkable decrease in the activity of − 7 bp/ + 270 bp construct compared with − 347 bp/ + 270 bp construct (Fig. 4B), implying that there were some positive elements in − 347 bp/− 7 bp region responsible for Runx2 activation in response to osteogenic induction.

Transcription factor binding site analysis of − 347 bp/− 7 bp region of Runx2 promoter indicated that there were 3 putative binding sites for AR (Additional file 1: Fig. S1). We further generated four constructs, − 347 bp/− 1 bp, − 327 bp/− 1 bp, − 62 bp/− 1 bp, and − 9 bp/− 1 bp (Fig. 4C). The results from transient reporter assay revealed that a significant decrease in − 62 bp/− 1 bp construct activity compared with − 327 bp/− 1 bp construct, and a significant decrease in − 9 bp/− 1 bp construct activity compared with − 62 bp/− 1 bp construct (Fig. 4D), suggesting that two AR binding sites within − 327 bp/− 1 bp region may be potential positive elements to modulate Runx2 expression.

To confirm the role of AR binding sites in Runx2 expression, we constructed plasmid vectors to overexpress wild type AR (AR-WT) and dominant negative AR (AR-DN) respectively and co-transfected each of them with − 327 bp/− 1 bp construct into BMSCs from 6-month-old mice. The − 327 bp/− 1 bp construct activity was enhanced significantly in response to AR-WT co-transfection compared with − 327 bp/− 1 bp construct alone (Fig. 4E). In contrast, the − 327 bp/− 1 bp construct activity was inhibited significantly in response to AR-DN co-transfection compared with − 327 bp/− 1 bp construct alone (Fig. 4E).

Further, we evaluated the binding of AR and HDAC6 on − 367 bp/ + 39 bp region of Runx2 promoter in BMSCs from young and aged mice with and without osteogenesis induction. The results from ChIP confirmed the binding of AR and HDAC6 on Runx2 promoter. AR binding in BMSCs from aged mice was much less than young mice (Fig. 4F). In response to osteogenesis induction, AR binding was enhanced in BMSCs from young and aged mice (Fig. 4F). Interestingly, HDAC6 binding on Runx2 promoter showed the opposite pattern relative to AR with and without osteogenesis induction (Fig. 4F), implying that there might be competitive binding HDAC6 and AR on Runx2 promoter. To confirm it, we overexpressed AR-WT and AR-DN in BMSCs from young and aged mice and then analyzed the binding of AR and HDAC6 on Runx2 promoter. The AR binding was enhanced significantly in response to AR-WT over-expression and inhibited significantly in response to AR-DN over-expression (Fig. 4G). In contrast, the HDAC6 binding was inhibited significantly in response to AR-WT over-expression and enhanced significantly in response to AR-DN over-expression (Fig. 4G). We further silenced HDAC6 expression by siRNA (Fig. 4H). The ChIP results revealed the inverse pattern between HDAC6 and AR binding on Runx2 promoter in BMSCs from young and aged mice, e.g. when HDAC6 binding was inhibited by siRNA, the enhancement of AR binding was observed (Fig. 4I).

### Runx2 expression activation in response to HDAC6 inhibition in BMSCs from aged mice

**Fig. 5:**
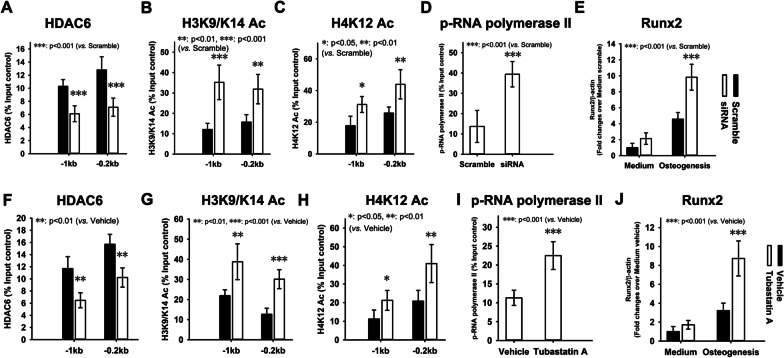
Runx2 expression activation in response to HDAC6 inhibition in BMSCs from aged mice. Lentivirus vector loading siRNA against HDAC6 was employed to knockdown HDAC6 expression in BMSCs from aged mice. Then ChIP assays were used to examine HDAC6 (**A**), acetylated H3K9/K14 (**B**)**,** acetylated H4K12 (**C**), and phosphorylated RNA polymerase II (**D**) occupancy in the Runx2 promoter regions. Quantitative RT-PCR was employed to measure Runx2 expression in BMSCs from aged mice in response to HDAC6 knockdown with and without osteogenic induction (**E**). ChIP assays were used to examine HDAC6 (**F**), acetylated H3K9/K14 (**G**)**,** acetylated H4K12 (**H**), and phosphorylated RNA polymerase II (**I**) occupancy in the Runx2 promoter regions in response to tubastatin A treatment. Quantitative RT-PCR was employed to measure Runx2 expression in BMSCs from aged mice in response to tubastatin A treatment with and without osteogenic induction (**J**). Data are shown as the mean ± SD. *p < 0.05, **p < 0.01, ***p < 0.001

Having observed Runx2 expression inactivation and HDAC6 accumulation on Runx2 promoter in BMSCs from aged mice, we then asked whether Runx2 expression could be activated through inhibiting HDAC6. To answer this question, we firstly silenced HDAC6 expression through siRNA. In response to siRNA-mediated HDAC6 knockdown, the ChIP results revealed a significant decrease in HDAC6 binding (Fig. 5A) and a significant increase in acetylated H3K9/K14 (Fig. 5B) and H4K12 (Fig. 5C) binding on Runx2 promoter in BMSCs from aged mice, compared with scramble control. Stronger p-RNA polymerase II binding was observed in response to HDAC6 silence in BMSCs from aged mice compared with scramble control (Fig. 5D). The BMSCs with HDAC6 knockdown were induced to undergo osteogenic differentiation in vitro. At week 1, total RNA was extracted and subjected to RT-PCR for Runx2. The results revealed significant increases in Runx2 expression in BMSCs from aged mice in response to HDAC6 knockdown compared with scramble control (Fig. 5E).

Also, tubastatin A, a specific inhibitor of HDAC6, was used to inhibit HDAC6. In response to tubastatin A-mediated HDAC6 inhibition, the ChIP results revealed a significant decrease in HDAC6 binding (Fig. 5F) and a significant increase in acetylated H3K9/K14 (Fig. 5G) and H4K12 (Fig. 5H) binding on Runx2 promoter in BMSCs from aged mice compared with vehicle control. Stronger p-RNA polymerase II binding on Runx2 promoter was observed in response to HDAC6 inhibition in BMSCs from aged mice compared with vehicle control (Fig. 5I). The BMSCs with tubastatin A treatment were induced to undergo osteogenic differentiation in vitro. The results revealed significant increases in Runx2 expression in response to HDAC6 inhibition compared with vehicle control in BMSCs from aged mice (Fig. 5J).

### In vitro osteogenic differentiation potential recovery of BMSCs from aged mice in response to HDAC6 inhibition

**Fig. 6:**
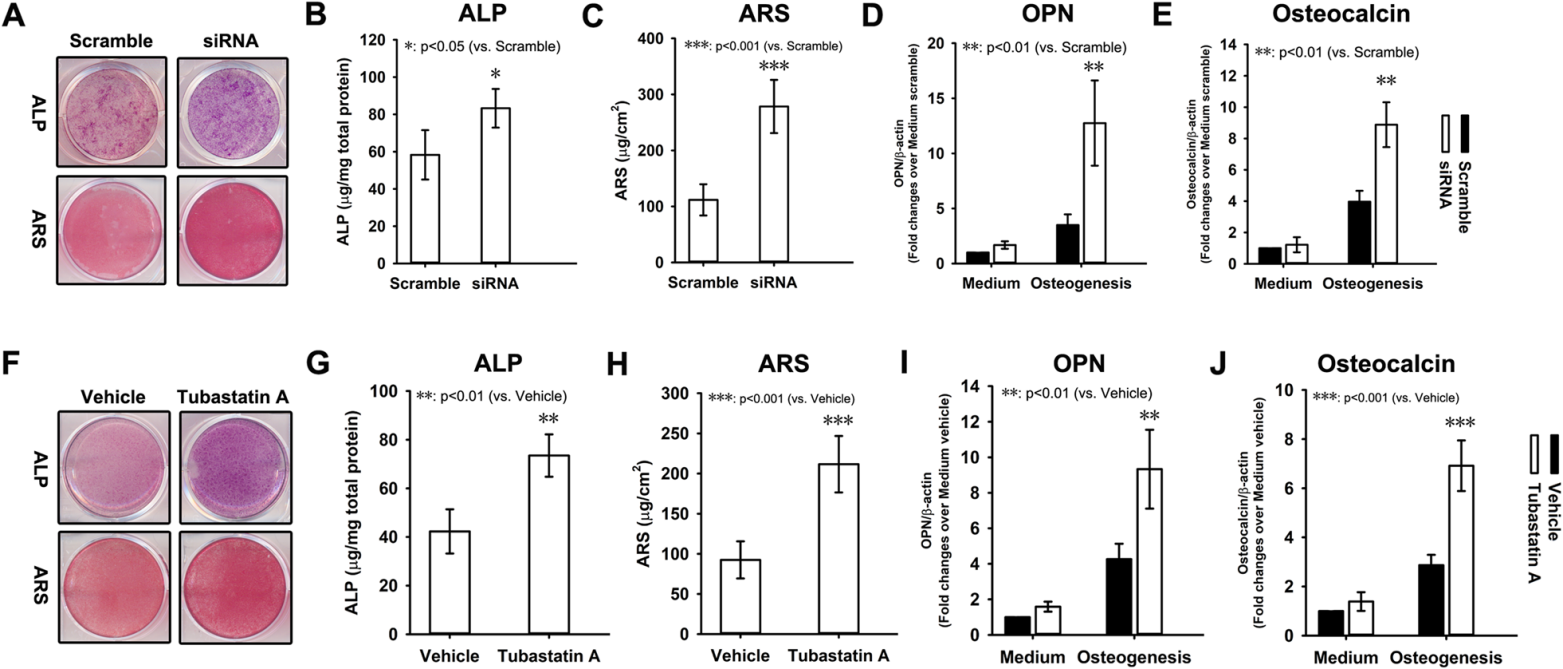
In vitro osteogenic differentiation potential recovery of BMSCs from aged mice in response to HDAC6 inhibition. Lentivirus vector loading siRNA against HDAC6 was employed to knockdown HDAC6 expression in BMSCs from aged mice. BMSCs were then induced to undergo osteogenesis differentiation. Alkaline phosphatase (ALP) and alizarin red staining (ARS) were performed at weeks 1 and 3, respectively (**A**). The quantification of ALP (**B**) and alizarin red (**C**) were also performed. At week 2, total RNA was extracted and subjected to quantitative RT-PCR analysis using primers for osteopontin (**D**) and osteocalcin (**E**). Tubastatin A was used to inhibit HDAC6 in BMSCs from aged mice. BMSCs were then induced to undergo osteogenesis differentiation. ALP and ARS were performed at weeks 1 and 3, respectively (**F**). The quantification of ALP (**G**) and alizarin red (**H**) were also performed. At week 2, total RNA was extracted and subjected to quantitative RT-PCR analysis using primers for osteopontin (**I**) and osteocalcin (**J**). β-actin was used as an internal control. Data were shown as the mean ± SD. *p < 0.05, **p < 0.01, ***p < 0.001

HDAC6 was silenced by siRNA in BMSCs from aged mice. Then the BMSCs were induced to undergo osteogenic differentiation in vitro. At week 1, the results from ALP staining revealed an enhancement in ALP activation in BMSCs from aged mice with HDAC6 knockdown compared with scramble control (Fig. 6A). At week 3, alizarin red staining in BMSCs from aged mice with HDAC6 knockdown was much stronger than scramble control (Fig. 6A). ALP (Fig. 6B) and ARS (Fig. 6C) quantification revealed the same pattern as staining. Total RNA was extracted at week 2 and subjected to quantitative RT-PCR. BMSCs from aged mice with HDAC6 knockdown showed higher levels of osteopontin (Fig. 6D) and osteocalcin (Fig. 6E) mRNA than scramble control in response to osteogenesis induction.

Also, tubastatin A was used to inhibit HDAC6 in BMSCs from aged mice. Then the BMSCs were induced to undergo osteogenic differentiation in vitro. At week 1, the results from ALP staining revealed an enhancement in ALP activation in aged BMSCs with HDAC6 inhibition compared with vehicle control (Fig. 6F). At week 3, alizarin red staining in BMSCs from aged mice with HDAC6 inhibition was much stronger than vehicle control (Fig. 6F). ALP (Fig. 6G) and ARS (Fig. 6H) quantification revealed the same pattern as staining. Total RNA was extracted at week 2 and subjected to quantitative RT-PCR. The BMSCs from aged mice with HDAC6 inhibition showed higher levels of osteopontin (Fig. 6I) and osteocalcin (Fig. 6J) mRNA than vehicle control in response to osteogenesis induction.

### The alleviation of age-related bone loss in aged mice in response to HDAC6 inhibition

17-month-old mice were administrated intraperitoneally with tubastatin A once every two days for 30 days. After 30 days, animals were euthanatized for micro-CT scan and serum biomarkers analysis.

**Fig. 7:**
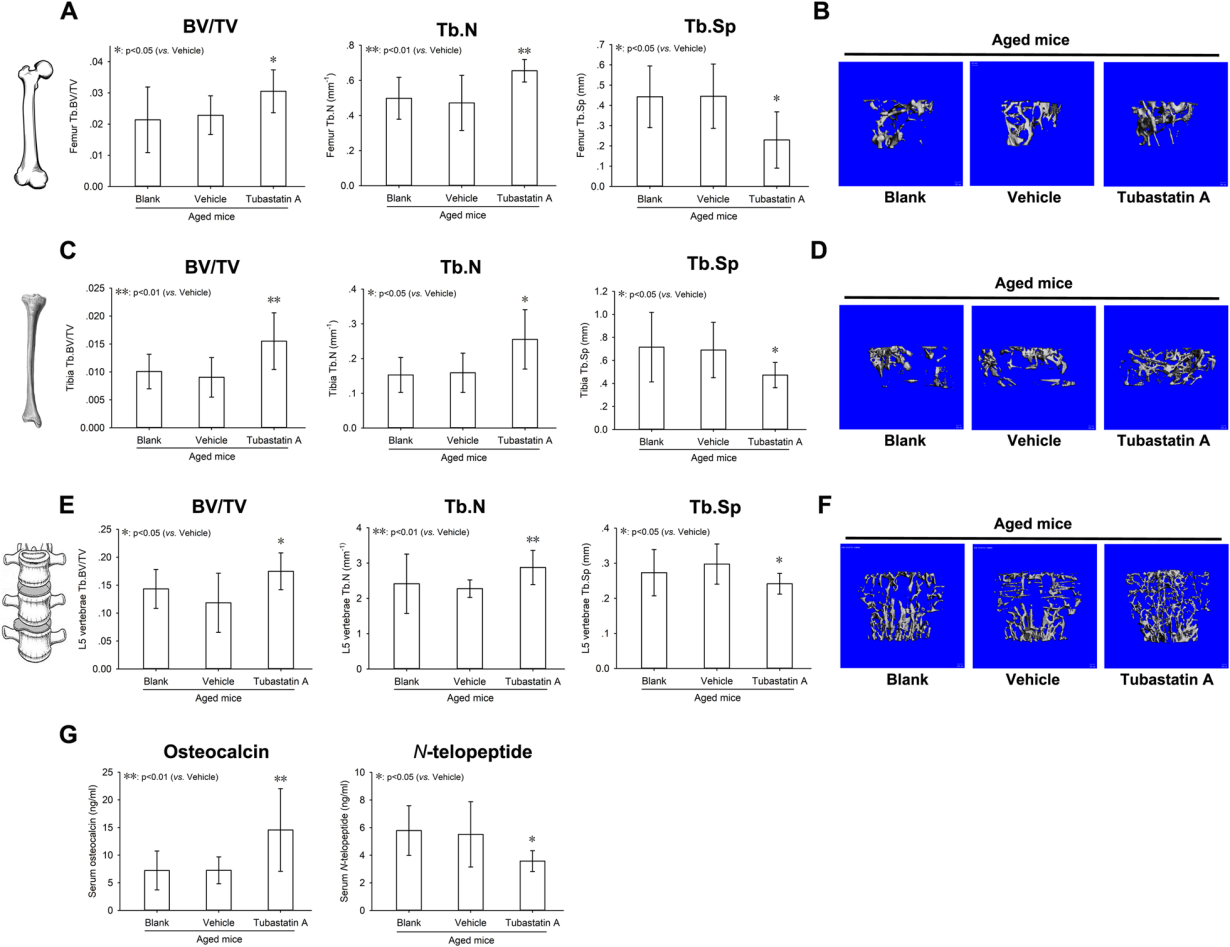
The alleviation of age-related bone loss of aged mice in response to HDAC6 inhibition. Tubastatin A, was administrated intraperitoneally to 17-month-old mice. After 30-day administration, femur (**A**), tibia (**C**) and L5 vertebrae (**E**) were harvested. Micro-CT was employed to evaluate the trabecular bone microstructure. Trabecular bone parameters including BV/TV, Tb.N, and Tb.Sp were quantified. Representative 3D reconstruction images of trabecular bone in the distal femur (**B**), proximal tibia (**D**), and L5 vertebrae (**F**) were displayed. Serum levels of bone turnover markers, osteocalcin and *N*-telopeptide, in aged mice with tubastatin A administration were determined by ELISA (**G**). Data were shown as the means ± SD. *p < 0.05, **p < 0.01, Tubastatin A versus vehicle

There was a 30% increase in BV/TV, a 32% increase in Tb.N and a 25% decrease in Tb.Sp in the distal femur of aged mice with tubastatin A administration compared with vehicle control (Fig. 7A). There was an 88% increase in BV/TV, a 37% increase in Tb.N and a 19% decrease in Tb.Sp in the proximal tibia of aged mice with tubastatin A administration compared with vehicle control (Fig. 7C). There was a 85% increase in BV/TV, a 24% increase in Tb.N and a 23% decrease in Tb.Sp in the L5 vertebrae of aged mice with tubastatin A administration compared with vehicle control (Fig. 7E). Micro-CT 3-D reconstruction images of trabecular bone also showed the same pattern (Fig. 7B, D, F).

A significant increase in osteocalcin was observed in serum from aged mice with tubastatin A administration compared with vehicle administration (Fig. 7G). Moreover, there was a significant decrease in serum *N*-telopeptide level in aged mice with tubastatin A administration compared with vehicle control (Fig. 7G).

It should be noted that tubastatin A administration did not cause abnormal changes of histological appearances of core organs including heart, liver, spleen, lung, and kidney in mice (Additional file 1: Fig. S2).

## Discussion

Generally laboratory mice achieve peak bone mass between five and six months of age and then is followed by a gradual decline as the mice age [14]. Therefore, 6- and 18-month-old C57BL/6 mice were employed to mimic age-related bone loss of human in the current study. The micro-CT scans revealed the significant loss of trabecular bone in femur, tibia, and L5 vertebrae in 18-month-old mice, suggesting that C57BL/6 mice is a reliable tool to investigate the pathophysiological mechanisms of age-related bone loss.

It is recognized that age-related bone loss is caused by decreased bone formation, rather than increased bone resorption as in postmenopausal osteoporosis. Defective bone formation may be due to a decrease in the amount or function of osteoblasts, or both. For example, BMSCs from aged C57BL/6 mice have remarkable impairment in their differentiation potential to osteoblasts in addition to decreases in the proliferation ability [15]. In the current study the serum analysis revealed no significant change in bone resorption marker *N*-telopeptide but a significant decrease in bone formation marker osteocalcin in aged mice compared with young mice, suggesting that bone formation was reduced significantly in aged mice and bone resorption was almost not influenced. At the cellular level, our results revealed a decrease in CFU-OB and an impairment of BMSCs in vitro osteogenic differentiation potential including the decreases of ALP production, calcium deposition, and the mRNA abundance of osteopontin and osteocalcin.

Till now, several important genes for bone formation have been identified [16–18], in which Runx2 plays an essential role [5]. In the current study, along with the attenuation of in vitro osteogenic differentiation potential of BMSCs from aged mice, the results revealed the inactivation of Runx2 expression in BMSCs from aged mice. In response to transcription activation, there are generally three sequential steps of structural changes of nucleosome: histone modifications, chromatin unfolding and histione loss [19]. Considering that histone modifications are closely related with gene transcription, we wonder whether the global levels of histone modifications are changed significantly if these modifications are not accumulated at up-regulated or down-regulated genes by chance. Our results from western blot showed that the global levels of acetylated H3K9/K14 and H4K12, HDAC1, 3, 4, 5, and 6 remain unchanged in BMSCs from young and aged mice. Therefore, these observations suggested that the changes in histone modification status in Runx2 are not fluctuation at the global level but gene-specific.

In the current study, the results from ChIP revealed the acetyl groups loss of H3K9/K14 and H4K12 and the accumulation of HDAC6 on the Runx2 promoter in BMSCs from aged mice. In contrast, no differences in HDAC1, 3, 4, and 5 were observed, implying that HDAC6 played an important role in histone modifications in Runx2 promoter. Further, when HDAC6 was inhibited by siRNA or inhibitor, Runx2 expression was improved, which confirmed the involvement of HDAC6 in Runx2 inactivation. Currently, 11 mammalian HDACs have been identified. These enzymes are subdivided into three classes [20]: (i) class I (HDAC1, 2, 3, and 8); (ii) class IIa (HDAC4, 5, 7, and 9) and class IIb (HDAC6 and 10) [21]; and (iii) class IV (HDAC11). While the ubiquitously expressed class I HDACs are located in the nucleus, the tissue specific expressed class II HDACs can shuttle between the nucleus and cytoplasm [20]. Osteogenic differentiation potential is improved by HDAC activity inhibition with small chemicals, which suggests a direct acetylation-dependent mechanism [22, 23]. However, in addition to direct mechanisms, further investigation revealed that HDAC inhibitors may change osteogenic differentiation potential indirectly by modulating MAPK signaling and gene expression profiles with the help of miRNAs [24]. Moreover, several HDACs have been shown to interact with Runx2. While HDAC1 and 3 have been shown to inhibit Runx2 expression [24–26], HDAC4, 5, 6, and 7 have ability to bind Runx2 and thus interfere with its transcriptional activation effects. Among the class II HDACs, HDAC6 has its unique characteristics. It is the only HDAC with two deacetylase domains and one ubiquitin-binding domain [21]. HDAC6 has two nuclear export signal motifs so that it exists predominantly in the cytoplasm, where it deacetylates non-histone proteins like α-tubulin [27]. To our knowledge, the current study showed for the first time that HDAC6 suppressed Runx2 expression through binding on Runx2 promoter and modifying histone acetylation status.

In the current study, competitive binding of HDAC6 and AR on Runx2 promoter was observed in BMSCs. Runx2 expression was modulated by both of them synthetically. These data suggest that gene expression is a complicated process including binding of transcription factors to the promoter of the target genes, the reorganization of a variety of nuclear factors surrounding chromatin and the transcriptional machinery construction at the promoter. Interestingly, in some cases, transcription factors which bind on various genes are not to activate transcription but to establish a scaffold to recruit associated proteins to the gene, which suppress transcription [9, 28].

Histone deacetylase inhibitors (HDIs) are small molecules which are able to inactivate HDACs by binding to their zinc-containing catalytic sites [29]. HDIs have been shown to induce a transient enhancement in osteoblast proliferation and survival, to induce osteogenic differentiation in vitro, to improve ALP production and expression of type I collagen, OPN, bone sialoprotein, and osteocalcin, and to increase the extracellular mineral deposition in several osteogenic cell lines and mesenchymal progenitor cells [23]. In the current study, tubastatin A, a specific HDAC6 inhibitor, was employed to inactivate HDAC6 in aged BMSCs. In response to tubastatin A treatment, Runx2 expression activation and in vitro osteogenic differentiation potential recovery of BMSCs from aged mice were achieved. More importantly, tubastatin A was administrated in vivo to aged mice. The results from micro-CT and serum bone turnover markers analysis revealed the alleviation of age-related bone loss of aged mice in response to tubastatin A administration. Ideal HDIs should target only one specific HDAC. In this sense, tubastatin A belongs to such an ideal HDIs since it was reported substantially more selective (over 1000-fold selectivity) to inhibit HDAC6 than other isozymes except HDAC8 (57-fold selectivity) [27].

Interesting data of current study came from the decrease in serum *N*-telopeptide level of aged mice in response to tubastatin A administration, although no difference in serum *N*-telopeptide level was observed between young and aged mice. These data imply that (i) HDAC6 might play an important role in the osteoclast functions; (ii) tubastatin A enhances bone mass of aged mice through not only increasing bone formation but also decreasing bone resorption. Several previous studies support our data. The Rho-mDia2-HDAC6 pathway is reportedly involved in osteoclast maturation by controlling podosome patterning through microtube acetylation in osteoclasts [30]. HDAC6 loss can inhibit osteoclast differentiation of RAW264.7 cells [31]. Specific inhibition of HDAC6 with tubastatin or ACY-1215 inhibited osteoclast funtions [32]. Moreover, we can not rule out the possibility that OPG/RANKL expression pattern in BMSC of aged mice is changed by tubastatin A-mediated HDAC6 inhibition, and then osteoclast differentiation is inhibited by OPG up-regulation and/or RANKL down-regulation of BMSC. Whatever, the molecular mechanism underlying in vitro and in vivo osteoclast functions modulated by HDAC6 is still an open question and needs further investigation.

## Conclusion

**Fig. 8:**
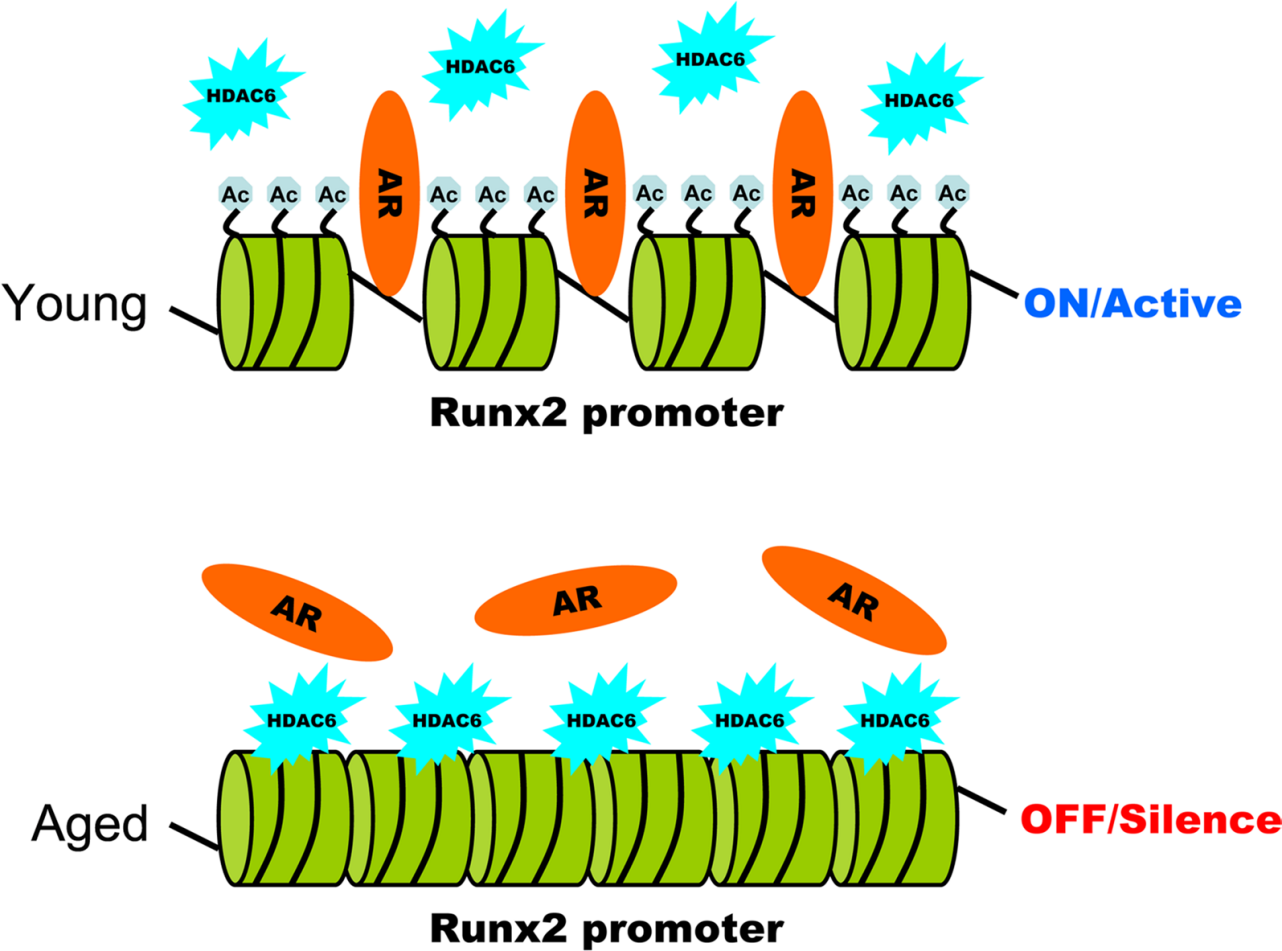
Model for HDAC6-mediated Runx2 expression modulation in BMSCs from young and aged mice. In BMSCs from young mice, HDAC6 is separated from Runx2 promoter so that the histone is hyper-acetylated and the chromatin remains incompact. AR has access to bind on Runx2 promoter to maintain Runx2 gene in “On/Active” status. In contrast, the histone in Runx2 promoter region is hypo-acetylated and the chromatin remains compact because of HDAC6 binding in BMSCs from aged mice. AR is not accessible to Runx2 promoter so that Runx2 expression remains “Off/Silence” status. Ac: Acetylation. AR: androgen receptor

In summary, the current study reported that HDAC6 accumulation and histone hypoacetylation on Runx2 promoter contributed to the attenuation of in vitro osteogenic differentiation potential of BMSCs from aged mice (Fig. 8). Through HDAC6 inhibition, we could activate Runx2 expression and osteogenic differentiation potential of BMSCs from aged mice and alleviate the age-related bone loss of aged mice. Our study will benefit not only for understanding the development fo age-related bone loss, but also for finding new therapies to treat senile osteoporosis.

## Supplementary Information

**Additional file 1.** Primer sequences used in quantitative RT-PCR (**Table S1**) and ChIP-qPCR (**Table S2**), Runx2 promoter sequence investigated in the current study (**Figure S1**) and histological evaluation of core organs of aged mice in response to Tubastatin A administration (**Figure S2**) are included in the supplementary information.

## Data Availability

The data that support the findings of this study are openly available in Mendeley at http://dx.doi.org/10.17632/s9bw7c4mgk.1.

## Abbreviations

BMSCs: Bone marrow stroma cells
Runx2: Runt-related transcription factor 2
ALP: Alkaline phosphatase
Opn: Osteopontin
Ocn: Osteocalcin
HAT: Histone acetyltransferase
HDAC: Histone deacetylase
AR: Androgen receptor
Micro-CT: Micro computed tomography
BV/TV: Bone volume/total volume
Tb.N: Trabecular bone number
Tb.Sp: Trabecular bone separation
ChIP: Chromatin immunoprecipitation
TSS: Transcription start site
WT: Wild type
DN: Dominant negative
HDI: Histone deacetylase inhibitor
