# Supplementary material for: HDAC6 inactivates Runx2 promoter to block osteogenesis of bone marrow stromal cells in age-related bone loss of mice
Source: Stem Cell Res Ther. 2021 Aug 28;12:484. doi: 10.1186/s13287-021-02545-w (PMC8403388; doi:10.1186/s13287-021-02545-w)
Supplement: Supplementary file 1 — Additional file 1. Primer sequences used in quantitative RT-PCR (Table S1) and ChIP-qPCR (Table S2), Runx2 promoter sequence investigated in the current study (Figure S1) and histological evaluation of core organs of aged mice in response to Tubastatin A administration (Figure S2) are included in the supplementary information. [file 13287_2021_2545_MOESM1_ESM.doc]

**Table S1: Primer sequences used in quantitative RT-PCR**

| **Gene** | **Primer sequences (5’-3’)** | **Product length (bp)** |
| --- | --- | --- |
| Runx2 | TTCAACGATCTGAGATTTGTGGG (F) | 221 |
|  | GGATGAGGAATGCGCCCTA (R) |  |
| Osteopontin | CCAGCAGCTCACACTGAAGA (F) | 207 |
|  | AAAAGTCTGTCGGAGTGCTGA (R) |  |
| Osteocalcin | GCTACCTTGGAGCCTCAGTC (F) | 71 |
|  | AGGGTTAAGCTCACACTGCT (R) |  |
| β-actin | GGCTGTATTCCCCTCCATCG (F) | 154 |
|  | CCAGTTGGTAACAATGCCATGT (R) |  |

**Table S2: Primer sequences used in ChIP-qPCR**

| **Gene** | **Primer sequences (5’-3’)** | **Product length (bp)** |
| --- | --- | --- |
| Runx2 (-1kb) | GAGTCGTGTCTATATAAACC (F) | 299 |
|  | TCTAGTGTCTAACTGTCTTT (R) |  |
| Runx2 (-0.2kb) | TGAAGTGGGGAGCCGAGGAC (F) | 407 |
|  | GCTCGCGGCGCCGTCGCTT (F) |  |

**Runx2 promoter sequence**

-1280bp

GGGCGCCGCCGCACGTTGTTTGATTTGTTTTGAAGGCTCAGAATTTGAGGCTGGTCGGAGACACCCACGTGCTTCTGATTCCCATCAGTATAATGATCGCCTCAGTTTGAGTCGTGTCTATATAAACCACAAAAACCTAATCATTAGAAATCCCAGCCTCCAAAAACCACATTTTAGGTAAAAAAACAAACAAAAAAAAAAAAAACAAACAAAAAAAAAACAAAACTGCTGCCGCTCCCCGCACTCCTTCATCTCTCAACCACAATCTTTTGGGGATTCCAAAATCGCCTCCCCCCATAACGCCGGGAAACAAAAACATAAATAAATAATTACTGAACTTTTTTTTAAACTTTTTATTTTTAACCCCCCAAAGTGGGGAGTGGCTAGAAAGACAGTTAGACACTAGACTTAGTCTGAGACGAAGACTACACTTAATAAAAGATTGGGAAATGAATGGATAACCGCTGTGTATTCCCCCTTTCCCAGCAAGCATTTTATCAACTTACAACTTATTTTAATTACCAAGCTGTCATTTTTTAGTTCTTTTTATTAAAAAAGCAACCCATCCTTATTCTCCAATAATGACAAGAAGGAATATATAAGAAAAGTGTCCAGACATCCGTTAGAGTTTCTAAAACTATTTTGACAAACACTCCTGTCACCCATTTTGGTGTCACCTAAGGGGGGGCACTCCCGAAGCCTGTAAAAACCTGCCCCCCGCCCCAACCAATGTGGGGTGGGGTTGGGGGCAGGGTGAAGAGTGCTCAGAAGTCAACGTGGAGACTTGAGAATTTCCCTTCCTCCTCTCCTGAAGTTAACAACGAAAAATTAACGCCAGTCGGAGCAGCCTGAGGCTCTCCCGCTTCTCAGCTTTAGCGTCGTCAGACCGAGAAGTGGTTCCCGGTCCTGAGGGTGAAGTGGGGAGCCGAGGACGCAGGCGGCGATGTCCTAGGCGGGGACCTCCTCCCTACAGCTTCGGGCGCCGAGCGAGCGCAGCGGCGCTTCTAGCGGCCGGCGGGCGGCGGCAGCGGCTGCGATCCGCAGGCTCCAGATCTGTCGCCCCGAGATCCGCTCTCCCCCCGCCCCCACTTACCTCCCGGCACCTTGAAACGCGAGGGGGGCCCGGGGCACTTTGCAAAGAGCAGGAGAGACGAGGCTGCGAGCTAGACGGCGGCGAAGGAGAGGGCGAGAGGAGAAGCCGGGGAAAGGAAGGACTCGGCGGCCGGAGGACTCGGAGCGCGCTGCCGGCGCGGGGAGCGCGCAGCGGCCTCGGAGGAGGAGGCGGAGGAGGCGGCGCGGGCAAGCGACGGCGCCGCGAGCTGGGCAGCCGCGCTCTGCTTGGCGGTGGCGGACAGCGAGGAGCCACACGCACCGCCGAGATGGACTGCTGAACCTGCGGGGCTCCACTACCGTACTGGAACCCGGAGCGGGGCGCCAGCGCACCCAGGACACGGTGCCCCAAGGGGCCCCTACTGCAAGCTGTTAACTTCAAGTCCCTTGCGGCGGCTGGGCCAAACACGCCCCCGCGTCCCGCTCGTTGCAGCCACCGC

+270bp

Transcription start site Putative AR binding site ChIP region

**Figure S1**

**Figure S1. Runx2 promoter sequence investigated in the current study.** The DNA sequence of -1280bp~+270bp around Runx2 transcription start site was shown. Transcription start site was labeled in red, putative AR binding sites were labeled in blue and ChIP regions were labeled in green.


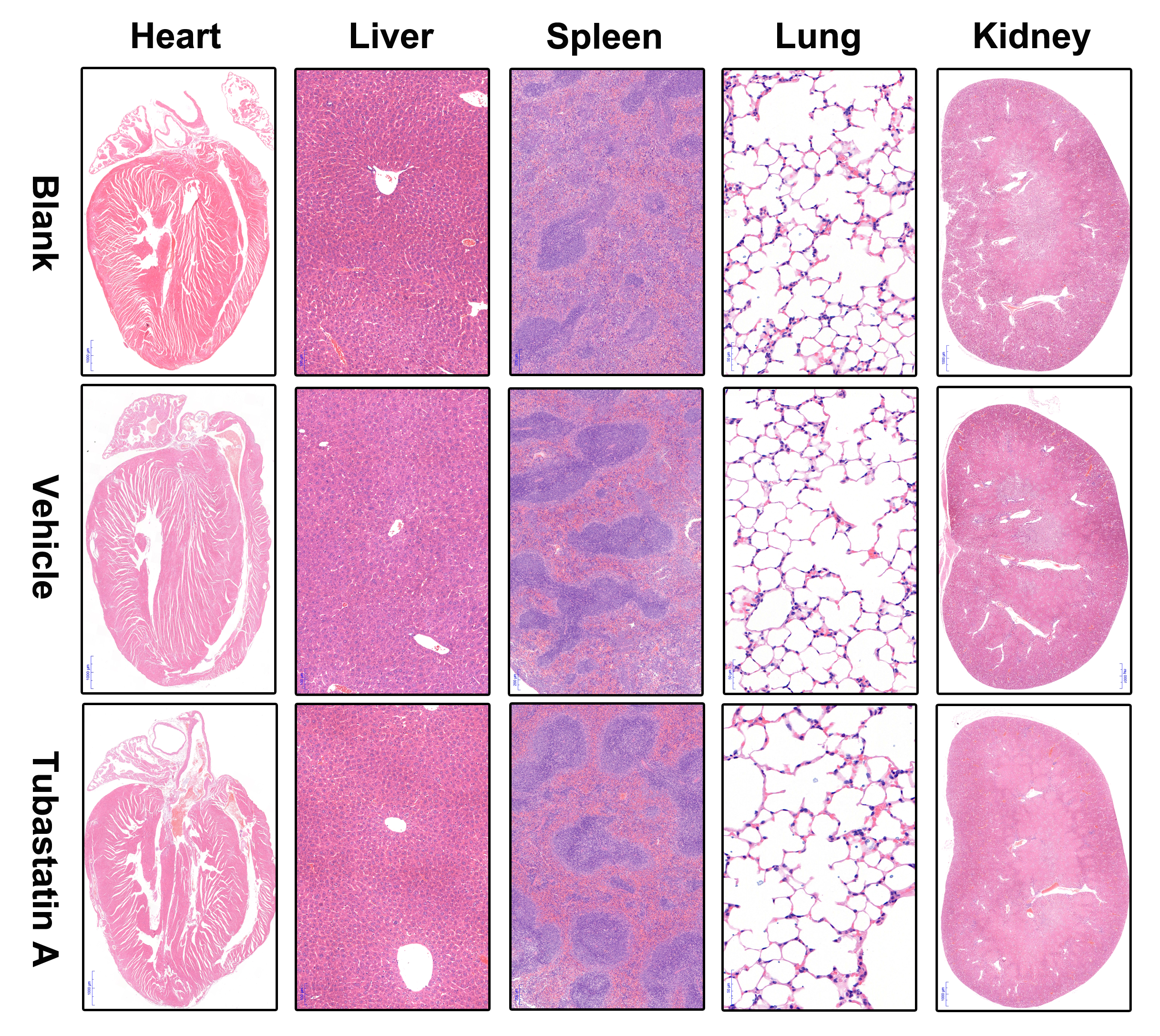


Figure S2

**Figure S2. Histological evaluation of core organs of aged mice in response to Tubastatin A administration.** Core organs including heart, liver, spleen, lung, and kidney were recovered from aged mice in response to Tubastatin A or vehicle (4% DMSO and 30% PEG300) administration. H&E staining was employed to evaluate the histological phenotype of these organs described above.
